# *In Vivo* Imaging of Pancreatic Islet Grafts in Diabetes Treatment 

**DOI:** 10.3389/fendo.2021.640117

**Published:** 2021-03-02

**Authors:** Dian R. Arifin, Jeff W. M. Bulte

**Affiliations:** ^1^Department of Radiology and Radiological Sciences, School of Medicine, Johns Hopkins University, Baltimore, MD, United States; ^2^Institute for Cell Engineering, School of Medicine, Johns Hopkins University, Baltimore, MD, United States; ^3^Department of Oncology, School of Medicine, Johns Hopkins University, Baltimore, MD, United States; ^4^Department of Chemical and Biomolecular Engineering, Whiting School of Engineering, Johns Hopkins University, Baltimore, MD, United States; ^5^Department of Biomedical Engineering, School of Medicine, Johns Hopkins University, Baltimore, MD, United States

**Keywords:** diabetes, imaging, islet transplantation, labeling, microcapsule, cell therapy

## Abstract

Transplantation of pancreatic islets has potential to offer life-long blood glucose management in type I diabetes and severe type II diabetes without the need of exogenous insulin administration. However, islet cell therapy suffers from autoimmune and allogeneic rejection as well as non-immune related factors. Non-invasive techniques to monitor and evaluate the fate of cell implants *in vivo* are essential to understand the underlying causes of graft failure, and hence to improve the precision and efficacy of islet therapy. This review describes how imaging technology has been employed to interrogate the distribution, number or volume, viability, and function of islet implants *in vivo*. To date, fluorescence imaging, PET, SPECT, BLI, MRI, MPI, and ultrasonography are the many imaging modalities being developed to fulfill this endeavor. We outline here the advantages, limitations, and clinical utility of each particular imaging approach.

## Introduction

Moment-to-moment regulation of blood glucose in type I diabetes (T1D) and severe type II diabetes (T2D) patients may be achieved by transplantation of pancreatic islets without the need for exogeneous insulin administration (1–3). Engrafted islets have been shown to stabilize blood glucose control, reduce the occurrence of hypoglycemia and lead to insulin independence albeit for only a short period of time. Indeed, phase 3 clinical studies on allogeneic islet transplantation in 48 T1D patients have been conducted at eight centers in North America. At 1 and 2 years post-treatment, 88 and 71% of patients successfully met the primary end point (HbA1c <7.0%), respectively (4). Islet therapy however suffers from autoimmune and allogeneic rejection as well as non-immune related factors, such as inadequate neovascularization of islet grafts. It has been a major challenge to elucidate the fate of islets after engraftment (5–7). At present, clinical trials rely on blood glucose levels and metabolic tests before and after transplantation to gauge the therapeutic performance (8, 9). These values do not accurately reflect the mass of successfully engrafted islets, and the survival and insulin secretion of islets *in vivo*. Many of these tests detect failed engraftment long after the majority of islet grafts have died and thus, the window to apply interventional therapy to save the implants has passed.

Recent work strives to develop imaging technology for non-invasive and clinically relevant interrogation of the distribution, number or volume, viability, and function of islet implants *in vivo*. The main challenges are the small size of islets (50–300 µm) and the lack of inherent contrast between islet grafts and the surrounding host tissue. Such limitations demand a high detection sensitivity of labeled islets. Histology, bioluminescence (BLI), and fluorescence microscopy imaging (FMI) have been widely utilized to assess transplanted islets in animal models but all three methods are not suitable for patient studies due to its invasiveness or lack of light penetration in deep tissues. PET, SPECT, magnetic resonance imaging (MRI), magnetic particle imaging (MPI), and ultrasonography are currently being developed for this specific purpose and, unlike BLI or FMI, have shown promising potential for clinical translation.

## Labeling of Islets

For *in vivo* imaging, islets or cells are typically labeled prior to transplantation with the exception of SPECT and PET radiolabeling. For BLI, islets or beta-like cells, such as INS-1E cells, can be transfected or transduced with luciferase gene commonly derived from firefly (10–17). Baculovirus has been used to mediate radiolabeling by ^125^I for SPECT imaging (18). However, genetic modification of cells raises serious concerns about adverse cell differentiation as well as their immunogenicity, leading to a long path of FDA approval if any. SPECT and PET tracers are designed to target beta cell-specific receptors and therefore label islet grafts *in situ* (19–22). For FMI, MRI, and MPI, islets are labeled *ex vivo* prior to transplantation by direct probe incubation. Here, nanoparticles can serve as a versatile platform for creating probes that can be detected by more than one imaging modality (10, 15, 23). The limiting factor in islet labeling is the total amount of probe that an islet can carry while maintaining preservation of islet viability and function as well as *in vivo* detection sensitivity. Henceforth, the development of optimum islet labeling probes and protocols remains an actively pursued field.

## Bioluminescence Imaging

**Figure 1:**
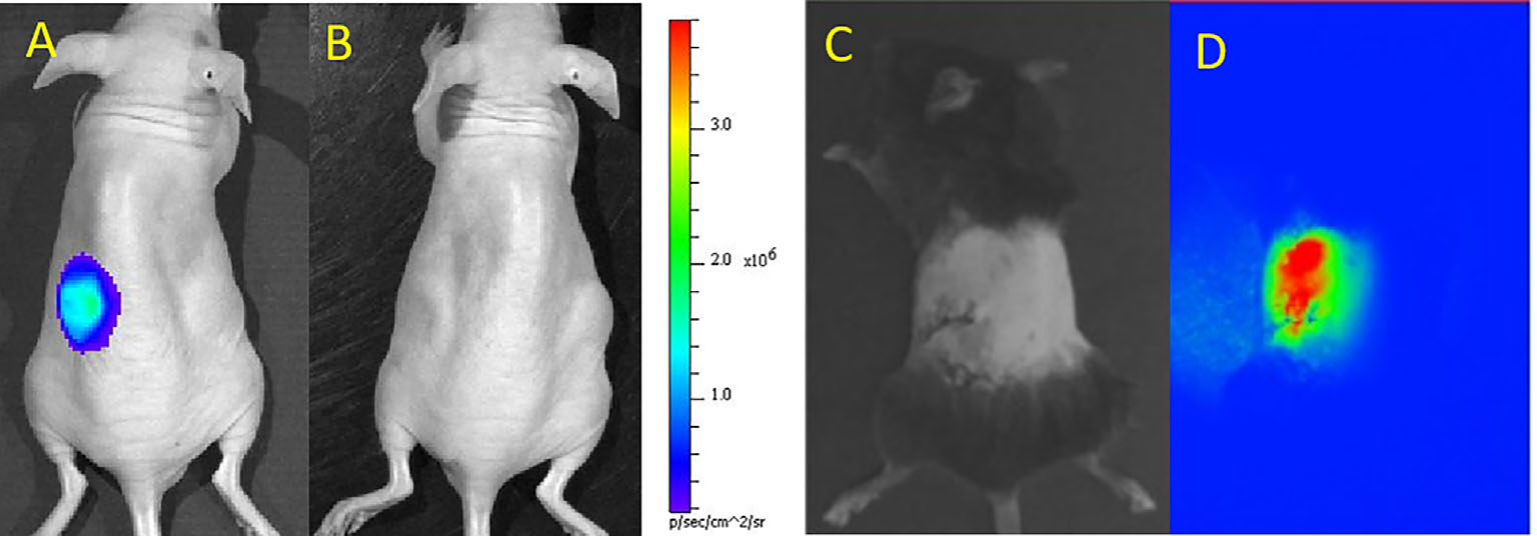
Bioluminescence (BLI) of luciferase-transduced islets at day 1 **(A)** and 4 **(B)** after subcutaneous transplantation in mice. BLI signals decreased as islets were progressively dying. Panels **(A, B)** are reproduced from (13). Bright field **(C)** and NIR fluorescence **(D)** microscopic images of indocyanine green/iron oxide labeled islets implanted under the kidney capsule of mice. Panels **(C, D)** are reproduced from (23).

Upon administration of the substrate D-luciferin to the islet recipients, the luciferase gene of labeled islets emits light. This photon emission is subsequently detected with a BLI instrument. BLI signals are registered as “hot spots”, therefore avoiding any confusion with background artifacts (**Figures 1A, B**). BLI is commonly overlaid on a regular photograph of the animal to provide anatomical context. This modality offers non-invasive quantitative imaging as the number of labeled islets linearly corresponds to photon emission, i.e., the intensity of the BLI signal (12). Since only live labeled islets produce photons, BLI can be used to monitor the survival of islet grafts, and to study the causes and timeline of islet death as well as graft distribution in a serial and quantitative manner.

However, deep tissues attenuate or scatter emitted photons measured by BLI (24) and compromise the readout. It is highly unlikely that the FDA will approve D-luciferin injection in patients in addition to issues related to genetic manipulation of cells and the expression of a xenogeneic protein (firefly luciferase). A typical D-luciferin injection dose for small rodents is no less than 150 µg/g body weight. Thus, the use of BLI has been confined to the study of cells and tissues in small rodents. BLI has been used to monitor the effects of 3D stem cell spheroids (13), poly(ethylene glycol)-encapsulants (14), mesenchymal stem cell-enriched scaffolds (16) or heparin-releasing silk fibroin scaffolds (17) on islet survival *in vivo* in rodents. The overall tolerance of islets for labeling them with perfluoro-15-crown-5-ether (PCE) emulsions for ^19^F MRI (10) or poly(lactic-co-glycolic acid) (PLGA) nanoparticles loaded with PCE and indocyanine green dye for ^19^F MRI and near-infrared (NIR) fluorescence imaging (15) has also been evaluated with BLI. Lastly, using TLR4-deficient mice and BLI, Gao et al. (11) showed that TLR4 activation mediated graft failure following intraportal islet transplantation.

## Fluorescence Microscopic Imaging

The limited depth of light penetration has largely limited FMI to examination of cells and tissues *ex vivo* or superficially. However, some recent developments have focused on *in vivo* imaging using FMI. Several potential clinical graft sites for islets have been explored, including subcutaneous chambers or intramuscular transplantation. As long as islets are transplanted superficially, FMI can be used to visualize the grafts. Nilsson et al. (25) implanted 20-30 human islets in the anterior chamber of the mouse eye, serving as an optical window for longitudinal monitoring with a high resolution 2-photon microscope. This study utilized transgenic *NOD*. (Cg)-*Gt(ROSA)26Sor*tm4-*Rag2*^−/−^ mice whose cells and tissues expressed a membrane-targeted tomato fluorescent protein to facilitate identification of the recipient and the donor tissues. Although the eyes have been considered to be an immunoprivileged site (due to the blood-ocular barrier), the clinical relevancy for this form of islet transplantation is a subject of debate. In a different study, iron oxide and human serum albumin-bound indocyanine green were encapsulated in clinically applicable PLGA nanoparticles. Three hundred syngeneic islets labeled with these nanoparticles and engrafted in the kidney capsule of C57/Bl6 mice could be well identified by NIR fluorescence microscopy (23) (**Figures 1C, D**).

## SPECT And PET Imaging

SPECT and PET imaging offer high detection sensitivity, deep tissue penetration and are well suited for clinical translation. Both modalities require the islets to be labeled with radionuclides, such as ^125^In, ^99m^Tc, ^68^Ga, or ^18^F. Hence, a drawback of SPECT and PET is radiation exposure, which should be avoided by pregnant or high-risk patients. In addition, the need for a cyclotron and/or radiochemical laboratory with trained personnel carries a high price tag. Due to the lack of anatomical information of SPECT and PET scans, both need to be co-registered with CT or MRI to provide tissue background anatomy. PET and SPECT signals appear as “hot spots” which can be used to quantify the number of radiolabeled islets. For clinical use, the sensitivity and spatial resolution of PET are approximately 2–3 orders of magnitude better than SPECT (26), but for small animal imaging, the resolution of SPECT is about 5-fold better than PET. However, SPECT radiotracers and equipment are less expensive than PET. SPECT radiotracers typically have a longer decay time than those of PET, thus allowing a longer observation period. On the other hand, a faster decay time may be desired to limit longer-term radioactive exposure.

**Figure 2:**
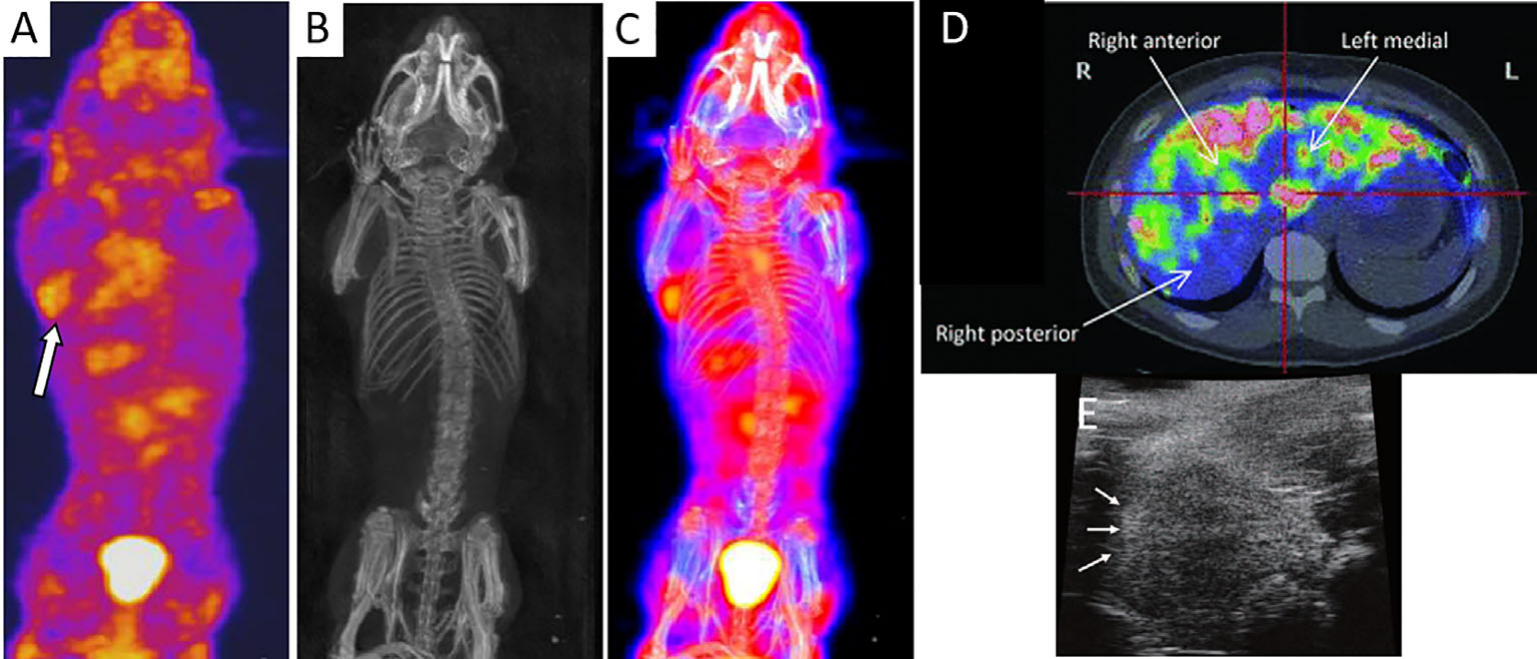
SPECT **(A)**, CT **(B)**, and an overlay of SPECT/CT images **(C)** of islets engrafted in the axillary cavity of mice after injection of radiolabel sodium ^125^I (arrow). Panels **(A–C)** are reproduced from (18). **(D)** Axial PET scan of ^18^F-fluorodeoxyglucose-labeled islets in the liver parenchyma of a T1DM patient. Islets can be seen as “hot spots.” Panel **(D)** is reproduced from (27). **(E)** HF-US image of islets under the renal capsules of a mouse which appeared as hyperechoic regions (arrows). Panel **(E)** is reproduced from (28).

For SPECT imaging, islets have been transduced with baculovirus vectors expressing the sodium iodide symporter for an enhanced uptake of sodium tracer agents (18). One to 8 h post-IV injection of 18.5 MBq of the radiotracer sodium ^125^I and 24 h after islet transplantation, SPECT/CT was able to visualize 2000 rat islet equivalents (IEQ) implanted in the axillary cavity of NOD-SCID mice (**Figures 2A–C**). Because of its low gamma energy, ^125^In is only suitable for small animal imaging, while ^123^In is the appropriate radionuclide for human imaging. Targeting receptors that are naturally present in islets is preferred instead of viral infection or genetic manipulation of islets. Demine et al. (29) developed a ^99m^Tc-labeled camelid antibody which bound specifically to alpha and beta islet cells by virtue of their dipeptidyl peptidase 6 expression. A high number of human islets (a cluster of 1,000 or 3,000 IEQ) subcutaneously engrafted in SCID mice could be detected by SPECT 1 h after probe administration (32–39 MBq per mouse) for 4 weeks post-transplantation. Willekens et al. (19, 20) used ^123^I-labeled iodobenzamide (58 MBq) to target D2 receptors expressed by beta cells for serial SPECT, and quantified syngeneic 1,000, 2,000 or 3,000 islet graft volume in the calf muscle of WAG/Rij rats for 10 weeks. The SPECT signal correlated linearly with the insulin‐positive graft volume, as confirmed by histology.

Studies to enable imaging lower numbers of islets are underway. Kroon et al. (21, 22) used ^111^In-exendin-3 to target glucagonlike peptide-1 receptors, specifically expressed on beta cells. Four weeks after transplantation of 400 or 800 syngeneic islets into the calf muscle of WAG/Rij rats, islet grafts could be visualized by SPECT at 1 h after systemic ^111^In-exendin-3 administration (15 MBq). *In vitro* assays validated again a linear relationship between the number of grafted islets and ^111^In-exendin-3 accumulation. Both studies by Willekens et al. and Kroon et al. demonstrated the potential for remote interrogation of islet graft quantity (volume or number) and distribution *in vivo*.

Five hundred or one thousand human islets engrafted *via* the portal vein in the liver of immunodeficient NOD/SCID mice could be visualized with microPET at 90 min after systemic injection of ~4 MBq ^68^Ga-DO3A-VS-Cys^40^-exendin-4, a radiolabeled glucagonlike peptide-1 receptor agonist (30). ^18^F-fallypride bound to D2/D3 receptors of islets and enabled PET imaging of 6,000 syngeneic Sprague–Dawley rat islet implants in the spleen at 120–180 min after intravenous (i.v.) injection of 28-37 MBq of probe (31). Furthermore, islet-like entities conjugated to avidin by means of a heparin scaffold could be visualized by PET in the liver of C57Bl/6 mice at 30 min after i.v. injection of 6 MBq [^68^Ga]Ga-DOTA-(PEG) (2)-biotin (32).

In a clinical trial, human islets were labeled with 20 MBq/ml of ^18^F-fluorodeoxyglucose for 60 min at 37°C. PET imaging of five T1DM patients receiving an intraportal infusion of 69,000 to 153,000 labeled, allogeneic islets revealed a 25% loss of islets during delivery (27). A heterogenous distribution of islet grafts with a marked concentration in small multifocal areas in the liver could be seen (**Figure 2D**). However, this technique was only applicable for 1 to 2 h post-implantation due to the radiotracer half-life of 110 min and short retention within the islets. In a different clinical study (33), eight patients with intraportal islet allografts (2–5 implant sessions of 210,000–800,000 IEQ each) underwent two PET sessions 8 months apart after i.v. administration of the serotonin precursor [^11^C]5-hydroxytryptophan (2–5 MBq/kg). However, the signals from liver uptake overwhelmed the signals from islet grafts although a few hotspots were seen in a few of the patients. In one patient, a change in hotspot uptake predicted graft function loss. With *in situ* labeling of entities that bind probes after i.v. injection, obfuscation of graft signal can occur by probe accumulation in other organs involved with metabolic clearance, such as the bladder, kidneys, and lungs, where potential toxic side effects need to be considered.

## Ultrasound Imaging

Ultrasonography is a fast, safe, and easy to operate imaging modality that involves no radiation. The device is portable, relatively inexpensive and available in most smaller clinics. This modality however suffers from a small window for imaging, an operator-dependent outcome and low detection sensitivity. Sakata et al. (28) employed high-frequency ultrasonography (HF-US) to monitor to monitor 200, 500, or 1,000 islets transplanted under the renal capsule of BALB/c mice (**Figure 2E**). Signals from syngeneic islets persisted while those from xenogeneic Sprague Dawley rat islets vanished by day 28, suggesting the ability of HF-US to report on islet survival. The islet volume calculated by HF-US correlated with the number as well as the metabolic function of islet grafts. In this study, islets were imaged without the use of labeling, avoiding potential adverse effects of labels. However, HF-US could only detect islet clusters, and not individual islets. In the clinic, ultrasound imaging revealed 230,000 autografted islets in one pancreatectomy patient as hyperechoic regions in the liver (34).

## Magnetic Resonance Imaging

MRI has the distinct advantage of being able to provide whole-body, detailed anatomical images of the subject with excellent contrast between soft tissues. Implanted labeled islets can be visualized along with the recipient anatomy using the same imaging system, avoiding any potential discrepancies that may be encountered in co-registration of images from two different systems as can be the case in SPECT/CT, PET/CT, or PET/MRI. The most common MRI technique involves the manipulation of water protons which are abundantly present in living tissue. ^1^H MRI contrast agents are categorized as T1-weighted or T2-weighted agents. T1-weighted agents enhance MRI signal and appear as bright signals or hyperintensities. In contrast, T2-weighted agents decrease MRI signal and appear as hypointensities.

Gadolinium (Gd)-based molecules are the most commonly used T1-weighted contrast agents. Gd is conjugated to biocompatible molecules/chelates to improve biocompatibility, circulation life and cell uptake. One thousand human and 500 murine BALB/c islets labeled with 50 mM Gd-HP-DO3A could be imaged after engraftment under the kidney capsule of mice as hyperintense entities (35). However, the risk of nephrogenic systemic fibrosis associated with dissociated free Gd has dampened the overall enthusiasm in using Gd-based contrast agents for clinical cell tracking (36).

The most widely used T2-weighted agent are superparamagnetic iron oxide nanoparticles (SPIOs), (previously) commercially available as ferumoxytol (Feraheme^®^), ferucarbotran (Resovist^®^), or ferumoxides (Feridex^®^ or Endorem^®^) (37–39). Islets or insulinoma-derived cells were labeled with SPIOs via direct incubation without any reported impairment of viability or function (23, 37–41). A number of techniques to improve the labeling efficiency, such as coating the surface of nanoparticles with heparin-protamine complex (37), heparin alone (42), poly-L-lysine (39), or phospholipids (39, 40), have been explored. Moreover, in an attempt to minimize or eliminate the toxicity of metal-based contrast agents, SPIOs have been embedded in biocompatible PLGA nanoparticles (23).

SPIO-labeled islets transplanted in the intraportal vein or kidney capsule of rodents were easily visualized as hypointensities from a few days up to 6 weeks post-surgery (23, 37, 39, 40, 43, 44). Hwang et al. (23) engrafted 300 syngeneic islets labeled with 250–500 µg/ml PLGA-coated SPIOs under the renal capsules of chemically induced diabetic C57Bl/6 mice which were imaged at 4.7T up to 4 weeks post-transplantation. In another study, islets were labeled with 800 mg/ml ferumoxytol, a clinically used ultrasmall (U)SPIO formulation, for 48 h *ex vivo* (37). Labeled syngeneic C57Bl/6 murine islet grafts could be visualized at 7T at 1 and 2 weeks after implantation under the kidney capsule or in the liver, with 300-600 IEQ given per mouse. Meanwhile, Ribeiro et al. created cationic phospholipid-coated SPIOs, also called magnetoliposomes (39, 40). MRI performed at 9.4T was able to visualize 200 labeled Sprague Dawley rat islets (containing ~35 ng of iron/islet) xenografted under the kidney capsule of C57Bl/6 mice for 4 days after engraftment. On the other hand, 50–1,000 labeled syngeneic islets could be detected much longer, up to 6 weeks post-transplantation in the same graft sites in Lewis rats.

When SPIOs are released from dying islets, the nanoparticles may linger inside the host tissue. As there are no means to differentiate T2-weighted signals originating from live *vs.* dead implants *vs.* free SPIOs, the accuracy of this modality to elucidate the survival of islet grafts is questionable. Furthermore, *in vivo* quantification of islet transplants from MRI signals is hampered by several factors (45, 46). It is impossible to discern between single islets and multiple islet clusters when the hypointense contrast becomes so high that no signal is left. Other confounding sources of negative contrast from intestinal air pockets and hemorrhage/endogenous iron deposits also generate T2-weighted effects. Furthermore, depending on the location of the graft, peristaltic movements may introduce motion artifacts.

**Figure 3:**
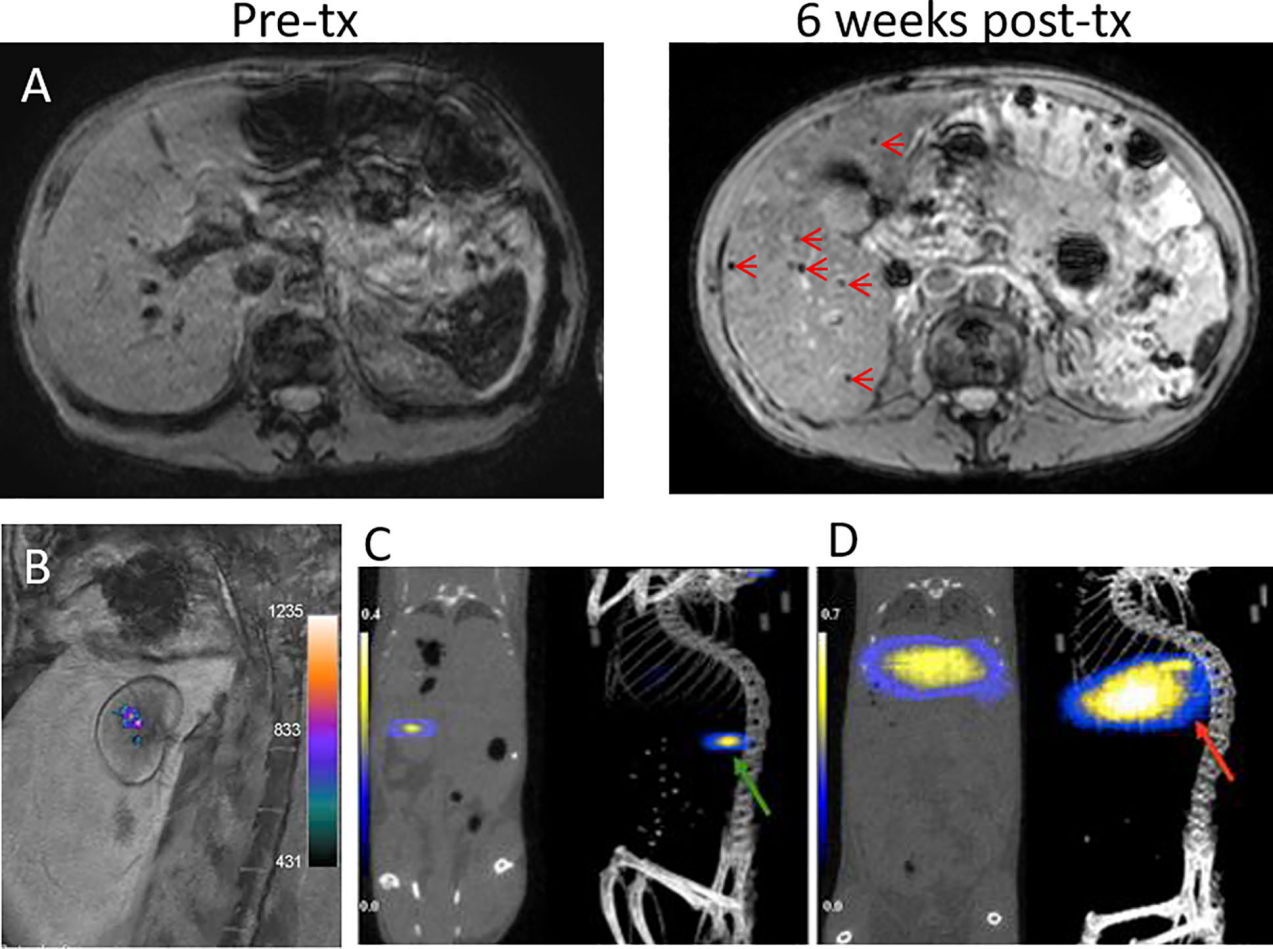
**(A)** 1.5T MRI of SPIO-labeled islets before and at 6 weeks after transplantation (tx) into the liver of a T1DM patient. Islet grafts are visible as hypointense spots (arrows). Panel **(A)** courtesy of Dr. T. Berney, originating from the same patient group reported by Toso et al. (45). **(B)** A 3T ^19^F/^1^H MRI overlay of PFPE-labeled human islets grafted under the renal capsule of rabbits. Panel **(B)** is reproduced from (47). ^19^F MPI/CT overlays of superparamagnetic iron oxide nanoparticle (SPIO)-labeled islets implanted under the kidney capsule (**C**, green arrow) and in the liver (**D**, red arrow) of mice. Panels **(C, D)** are reproduced from (48).

Indeed, clinical trials of SPIO-labeled islets infused into the liver via the intraportal vein have been performed with varying outcomes. The first demonstration on a safe use of SPIO-labeled islets in patients was reported in 2008 by Toso et al. (45). Here, islet allogeneic implants could be detected at 1.5T as hypointensities scattered in the liver parenchyma in three out of four T1D patients at 5 days, 6 weeks, and 6 months after transplantation (**Figure 3A**). Each patient received two deliveries of 50,000–375,000 IEQ per session, labeled with 280 µg iron/ml Resovist^®^. This study was followed by Saudek et al. (46) using Resovist^®^-labeled islets in eight T1DM patients. Each patient underwent one or two engraftments of 73,00–687,000 total IEQ (140 µg iron/ml), which was followed at 3T for 24 weeks post-delivery. About a 60% MRI signal decrease was observed within 1-week post engraftment, suggesting early rejection or erroneous transplantation, but hypointense signals persisted up to 24 weeks.

^19^F MRI directly detects tracers carrying ^19^F nuclei. Fluorine labeling of islets did not appear to affect their viability and function (47, 49). Unlike T1-weighted or T2-weighted MRI, ^19^F labeled-islets are visible as “hot spots” (50), very much alike PET and SPECT tracer imaging. Due to the scant amount of ^19^F atoms naturally present in the body, ^19^F MRI practically has no background signal interference. The images are overlaid on anatomical images first acquired by ^1^H MRI. ^19^F MRI enables non-invasive quantification of islet implants since the signal intensity correlates linearly to the number of labeled islets. Barnett et al. (47) was the first who labeled human islets with perfluoropolyether (PFPE) emulsions for ^19^F MRI visualization of 10,000 IEQ xenografted under the kidney capsules of rabbits using a 3T clinical scanner (**Figure 3B**). Moreover, *in vitro* tests demonstrated accurate quantification of labeled islets from ^19^F MRI signals. The signal from the rabbit study corresponded to 14,200 μg PFPE. Liang et al. (10) labeled Wistar rat islets and beta cell-like INS-1E cells with perfluoro-15-crown-5-ether (PCE) emulsions. Two hundred rat islets (~1 X 10^15 19^F atoms/islet) or 1 million INS-1E cells (~0.5–1 X 10^13 19^ F atoms/cell) subcutaneously xenografted in the right thigh of Swiss nude mice could be visualized with ^19^F MRI at 9.4T for 70 and 21 days post-delivery, respectively. In a study by Galisova et al., biocompatible PLGA nanoparticles were used as an encapsulant for fluorine labeling of islets, yielding ^19^F atom content of ~5 X 10^14^ per islet (15). ^19^F MRI signals from 1,000 to 3,000 subcutaneously implanted syngeneic islets in Lewis rats could be captured using 4.7T Bruker Biospin MRI up to 14 days after transplantation. However, the low detection sensitivity of ^19^F MRI demands high loading of tracers which remains challenging.

## Magnetic Particle Imaging

MPI is an emerging technique that directly detects the magnetization of iron oxide nanoparticles in a quantitative fashion. At present, there is only a single report on MPI of transplanted islets labeled with dextran-coated Ferucarbotran SPIO (~0.1 µg Fe/IEQ) (48). 9.4T MPI showed the presence of labeled baboon islets xenografted in the liver and under the kidney capsule of NOD SCID mice as “hot spots” (**Figures 3C, D**), combined with CT for anatomical imaging. Quantification of MPI signal revealed signal loss at 2 weeks post-engraftment which coincided with the timeline of islet graft destruction, thus demonstrating a capacity to be a surrogate marker of islet destruction similar to that previously seen with MRI. Despite MPI’s potential for clinical use, its development is at an infant stage and requires extensive work for bringing the system and MPI tracers to into the realm of clinical translation (51).

## Multimodal Imaging

**Figure 4:**
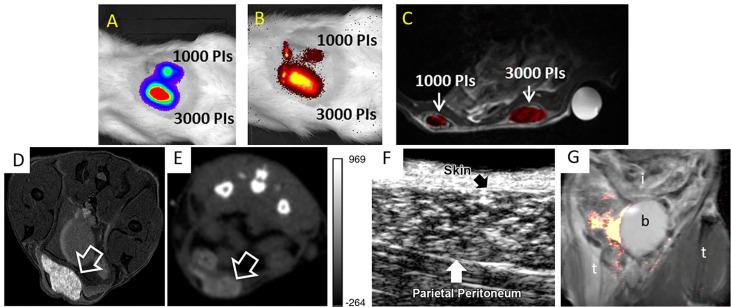
Bioluminescence (BLI) **(A)**, NIR imaging **(B)**, and an overlay of ^19^F/^1^H MRI **(C)** of multi-labeled islet grafts in rat abdomen. Panels **(A–C)** are reproduced from (15). PI = pancreatic islets. **(D)** T1-weighted ^1^H MRI **(D)**, CT **(E)**, and ultrasound **(F)** images of microencapsulated βTC-6 insulinoma cells (arrow) labeled with gadolinium/gold nanoparticles in the subcutaneous pouch of a mouse. Panels **(D–F)** are reproduced from (52). **(G)** ^19^F MRI of fluorinated microcapsules in mouse peritoneal cavity. t = thigh; b = bladder; i = intestines. Panel **(G)** is reproduced from (53).

As each imaging tool possesses its own advantages as well as limitations, researchers have created multi-modal probes in order to combine different imaging modalities within a single labeling platform. Liang et al. (10) labeled rat islets and INS-1E cells with PCE emulsions for ^19^F MRI and firefly luciferase gene for BLI. The authors reported that ^19^F MRI provided high resolution images of the distribution of engrafted cells while BLI measured the survival of transplants overtime. As mentioned above, Hwang et al. (23) synthesized PLGA nanoprobes encapsulating SPIOs and fluorescent dye indocyanine green to label murine islets. Both T2-W MRI and NIR imaging could visualize labeled islet implants in mice, presenting simultaneous clinical (MRI) and research-level (NIR imaging) utilization. Galisova et al. (15) also employed PLGA nanoparticles to carry fluorine and indocyanine green to label islets extracted from transgenic rats expressing the luciferase protein, and hence enabled trimodal monitoring by ^19^F MRI, NIR imaging, and BLI (**Figures 4A–C**).

## Microencapsulation for Imaging and Immunoprotection of Islets

A different approach for imaging islet grafts is to add imaging agents during immunoprotective microencapsulation of islets (54–56). In this design, the microcapsules are labeled instead of islets, thus avoiding direct manipulation of cells. A high concentration of imaging agents can be achieved here, higher than what can be obtained with labeling islets themselves, significantly augmenting detection sensitivity to the level of single capsule/islet detection. So far, alginate microencapsulated islets have been labeled with SPIOs (for T2-weighted ^1^H MRI) (57–59), fluorinated emulsions (for ^19^F MRI) (53, 60, 61), barium and bismuth (for CT) (62), gadolinium/gold nanoparticles (for T1-weighted ^1^H MRI, CT and ultrasonography) (52) and SPIO/gold nanoparticles (for T2-weighted ^1^H MRI, CT and ultrasonography) (54, 63) (**Figures 4D–G**). Furthermore, due to their intrinsic radiopacity, microcapsules synthesized using Ba^2+^ ion cross-linker could be visualized with micro-CT without the need of further labeling (64).

Upon the rupture of ^19^F MRI-visible microcapsules or fluorocapsules, the ^19^F MRI signal disappeared due to the release of fluorine into host tissues in a T1D model of NOD/Shiltj mice (53). This new approach may be used to non-invasively investigate the loss of immunoprotection imparted by disintegrating microcapsules and the subsequent rejection and death of islet grafts as was confirmed by BLI in this study.

## Future Outlook

Long-term labeling stability, toxicity of labels and detection sensitivity are issues that need to be addressed for successful translations of islet imaging techniques to the clinics. At present, the majority of imaging modalities only detect the localization and distribution of islet grafts not long after transplantation. Better methods to elucidate islet viability, function as well as the number or volume of islets throughout the treatment period will provide a more complete information on the fate of islets after delivery. Despite recent progress in the field of *in vivo* islet imaging, much work remains to be done to further improve the precision and efficacy of image-guided islet therapy.

## Author Contributions

DA performed a literature search and wrote the manuscript. JB edited the manuscript. All authors contributed to the article and approved the submitted version.

## Funding

The authors are supported by NIH R01 DK106972.

## Conflict of Interest

The authors declare that the research was conducted in the absence of any commercial or financial relationships that could be construed as a potential conflict of interest.
